# Supplementary figures and images for: Erbin interacts with NHERF1 and Ezrin to stabilize a membrane ErbB2 signaling complex in HER2-positive breast cancer
Source: Breast Cancer Res. 2025 May 19;27:85. doi: 10.1186/s13058-025-02025-6 (PMC12090624; doi:10.1186/s13058-025-02025-6)

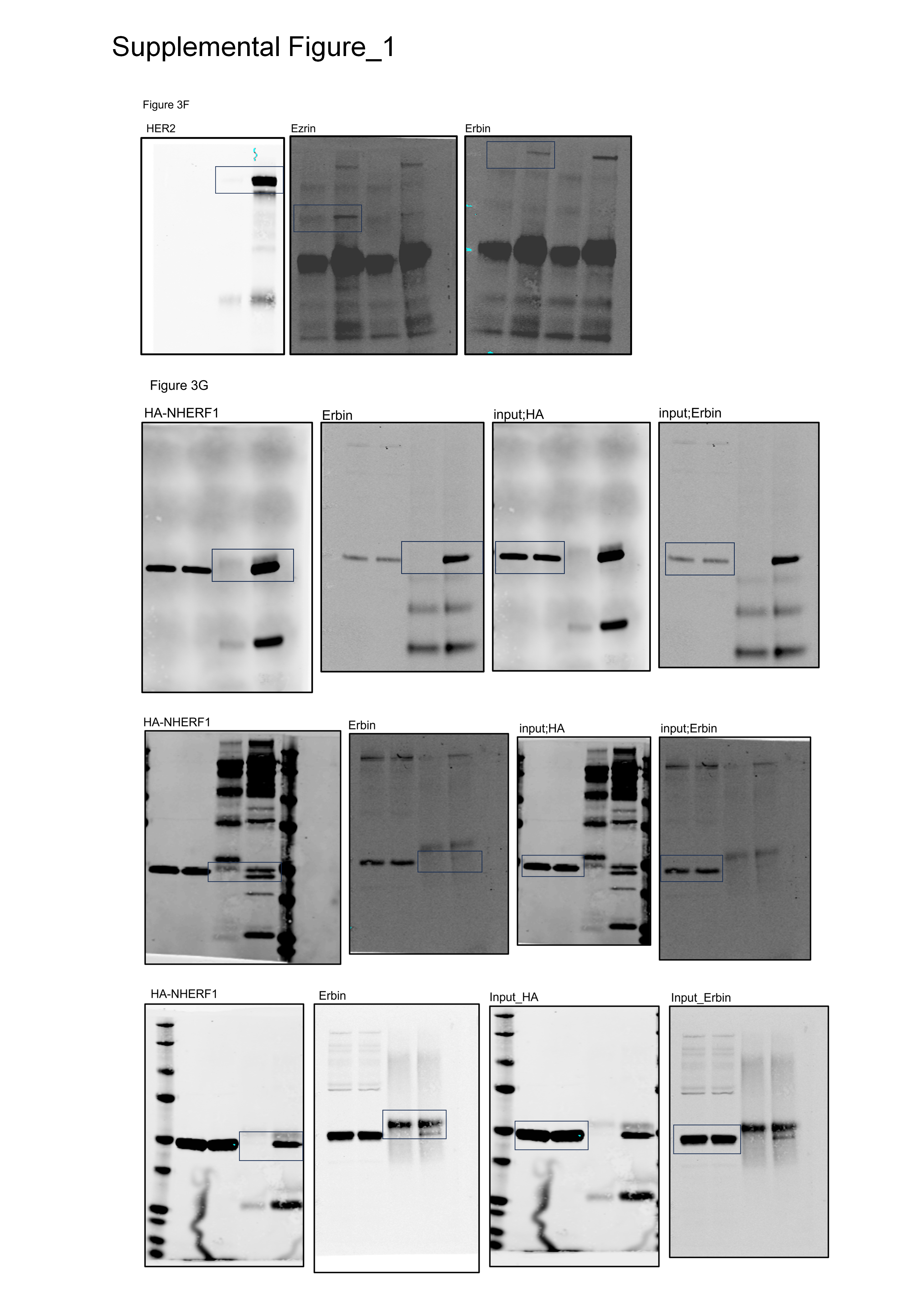

Supplement: Supplementary file 1 — Supplementary Material 1 [file 13058_2025_2025_MOESM1_ESM.tif]

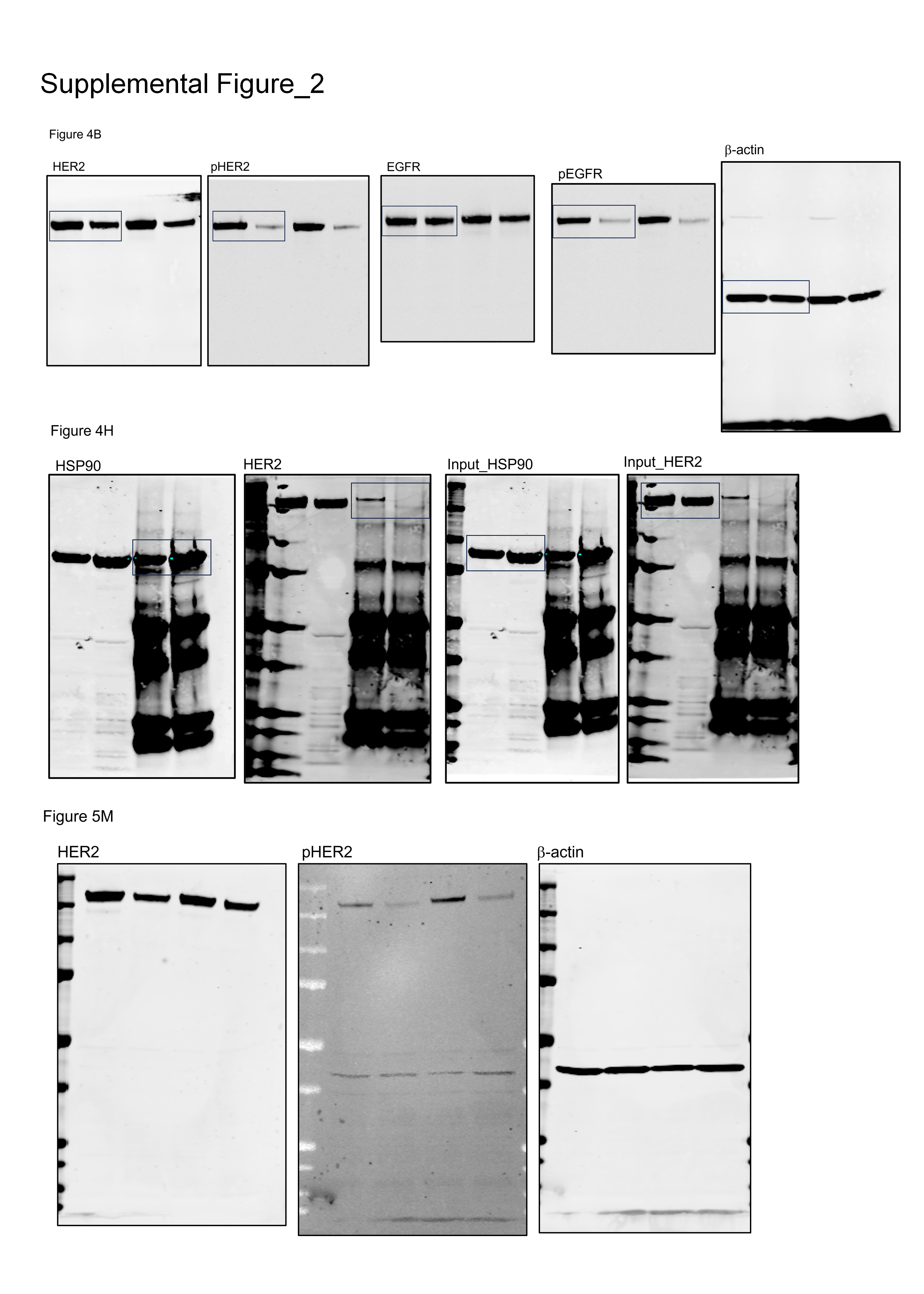

Supplement: Supplementary file 2 — Supplementary Material 2 [file 13058_2025_2025_MOESM2_ESM.tif]
